# Effects of silver doping at the A-site on the structure, surface morphology, and magnetic behavior of La_1−*x*_Ag_*x*_SrMn_2_O_5+*δ*_ (*x* = 0.1 and 0.2)

**DOI:** 10.1039/d5ra02729b

**Published:** 2025-07-02

**Authors:** Sonia Soltani, Mokhtar Hjiri, Abdullah M. Aldukhayel, Manel Essid, Anouar Jbeli, Nouf Ahmed Althumairi

**Affiliations:** a Department of Physics, College of Science, Qassim University Buraidah 51452 Saudi Arabia S.soltani@qu.edu.sa; b Department of Physics, College of Sciences, Imam Mohammad Ibn Saud Islamic University (IMSIU) Riyadh 11623 Saudi Arabia; c Department of Physics, College of Science, Majmaah University 11952 Al-Majmaah Saudi Arabia; d Chemistry Department, College of Science, King Khalid University (KKU) P.O. Box 9004 Abha 61413 Saudi Arabia

## Abstract

This study explores the impact of silver substitution at the A-site on the structural, morphological, and magnetic properties of La_1−*x*_Ag_*x*_SrMn_2_O_5+*δ*_ compounds, specifically for doping levels *x* = 0.1 and 0.2. The samples were synthesized using a conventional solid-state reaction method. X-ray diffraction (XRD) analysis confirmed that both compositions crystallize in a single-phase orthorhombic structure with the *Pnma* space group. Surface morphology and grain distribution were characterized *via* scanning electron microscopy (SEM), revealing microstructural changes associated with Ag doping. Temperature-dependent magnetization measurements indicate that Ag substitution leads to a reduction in overall magnetization, while the Curie temperature (*T*_p_), associated with the ferromagnetic-to-paramagnetic transition, remains essentially unaffected. This transition was identified from the derivative of the magnetization *versus* temperature curves. At lower temperatures, subtle anomalies in the magnetization data suggest the possible emergence of short-range antiferromagnetic interactions or charge ordering tendencies, without evidence of a distinct AFM phase transition. Magnetocaloric properties were also evaluated, showing notable variations in magnetic entropy change (−Δ*S*_m_) near the transition temperatures. The compounds exhibit relatively high values of relative cooling power (RCP), highlighting their potential for magnetic refrigeration applications.

## Abstract 2

This study explores the impact of silver substitution at the A-site on the structural, morphological, and magnetic properties of La_1−*x*_Ag_*x*_SrMn_2_O_5+*δ*_ compounds, specifically for doping levels *x* = 0.1 and 0.2.

## Graphical abstract

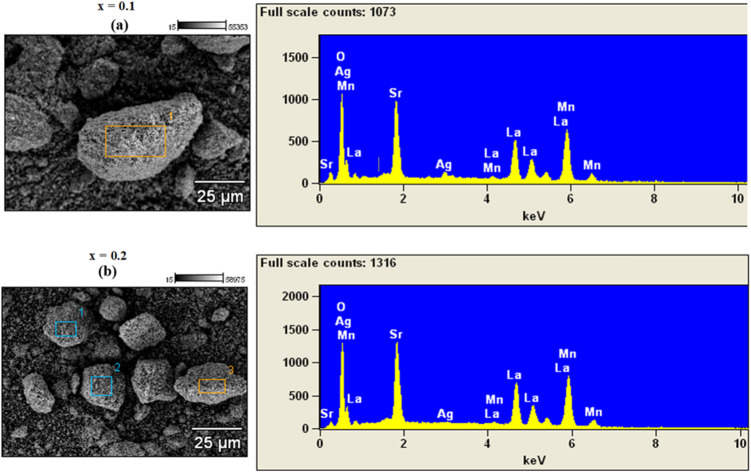

## 1. Introduction

The rapid advancement of science and technology over recent decades has led to an urgent demand for multifunctional materials that can address a variety of global challenges, including energy sustainability, environmental remediation, and green manufacturing.^1–30^ Innovative materials research spans a diverse spectrum from eco-compensatory watershed management and carbon footprint mitigation in forestry products^31–45^ to novel nanomaterials for heavy metal pollutant capture and advanced geopolymers for soil remediation.^46–70^ The convergence of environmental science, materials chemistry, and applied physics has accelerated the development of high-performance compounds that can integrate energy efficiency, environmental safety, and scalable manufacturing.^71–85^ For example, engineered materials exhibiting tailored electromagnetic responses, corrosion resistance, and tunable phase behavior are essential for applications ranging from magnetic refrigeration and energy harvesting to advanced catalysis and sensor technologies.^86–91^

Within the realm of functional materials, perovskite-type oxides have garnered significant interest due to their remarkable structural flexibility and rich spectrum of electronic, magnetic, and catalytic properties.^92–100^ Their tunability through compositional modifications and doping strategies enables precise control over crystallographic phases and physical behaviors, rendering them suitable for emerging technologies such as magneto resistive devices, spintronics, and environmentally benign refrigeration systems.^101–111^ The interplay between crystal structure, defect chemistry, and microstructural morphology determines their overall performance, necessitating advanced synthesis and characterization techniques, including solid-state reaction methods, X-ray diffraction, and electron microscopy.^112–120^ These techniques facilitate understanding how grain boundaries, oxygen vacancies, and dopant distribution influence magnetic ordering, electronic transport, and phase stability.^121–126^

Perovskite manganites, with the general formula Ln_1−*x*_A_*x*_MnO_3_ (Ln = La, Pr, Nd; A = Sr, Ca, Ba, Ag), represent a particularly compelling class of materials where charge, spin, and lattice degrees of freedom interact intricately to give rise to phenomena such as colossal magnetoresistance, metal–insulator transitions, and complex magnetic phase diagrams.^127,128^ The substitution of A-site cations modulates the Mn^3+^/Mn^4+^ ratio, directly impacting the double-exchange mechanism that governs ferromagnetic metallicity, while also affecting electron–phonon coupling linked to Jahn–Teller distortions.^129,130^ This delicate balance results in temperature-dependent magnetic transitions, including ferromagnetic, antiferromagnetic, and charge-ordered states, which can be fine-tuned by doping level and oxygen stoichiometry.^131,132^ Moreover, these manganite's exhibit significant magnetocaloric effects near their Curie temperatures, positioning them as promising candidates for energy-efficient magnetic refrigeration technologies that offer environmentally friendly alternatives to conventional gas compression cooling.^133,134^

Among the various dopants, silver (Ag) substitution at the A-site is of particular interest due to its distinctive ionic radius and electronic properties, which influence microstructure, magnetic interactions, and grain boundary effects differently compared to more commonly used divalent cations.^135,136^ Although silver doping has shown potential in enhancing magnetoresistance and modifying phase stability, systematic studies on its effects on La_1−*x*_Ag_*x*_SrMn_2_O_5+*δ*_ compounds remain scarce. Additionally, the impact of silver incorporation on magnetocaloric properties and low-temperature magnetic ordering phenomena such as antiferromagnetism or charge ordering warrants thorough investigation to better understand the underlying mechanisms and optimize functional performance.

This study aims to fill these gaps by synthesizing La_1−*x*_Ag_*x*_SrMn_2_O_5+*δ*_ (*x* = 0.1, 0.2) compounds *via* conventional solid-state reaction and characterizing their structural, morphological, and magnetic properties using X-ray diffraction, scanning electron microscopy, and magnetization measurements. We further evaluate their magnetocaloric potential through analysis of magnetic entropy change and relative cooling power, thereby contributing to the broader goal of developing advanced manganite materials for energy-efficient cooling and multifunctional applications. Our findings offer new insights into the role of silver doping at the A-site in tuning magnetic transitions and microstructure–property relationships in complex oxide systems, with implications extending across materials science and sustainable technology sectors.

## 2. Synthesis and characterization

### 2.1. Sample preparation

Polycrystalline samples of La_1−*x*_Ag_*x*_SrMn_2_O_5+*δ*_ (abbreviated as LASMO), with doping levels *x* = 0.1 and *x* = 0.2, were synthesized using the conventional solid-state reaction method, a widely used approach for preparing complex oxide ceramics due to its simplicity, scalability, and effectiveness in forming thermodynamically stable phases. This method allows for precise control over stoichiometry, is cost-effective, and suitable for preparing bulk quantities of high-purity compounds. It is particularly effective in promoting solid-state diffusion between precursor oxides and carbonates when subjected to high-temperature treatment. High-purity precursors La_2_O_3_, SrCO_3_, MnO_2_, and Ag_2_CO_3_ (≥99.9%) were used as starting materials. To ensure chemical stability and prevent moisture contamination, La_2_O_3_ was pre-calcined at 700 °C for several hours to remove any adsorbed water or carbonate impurities that could affect stoichiometry. Appropriate stoichiometric amounts of each precursor were calculated to match the nominal composition of La_1−*x*_Ag_*x*_SrMn_2_O_5+*δ*_. The powders were thoroughly ground and homogenized using an agate mortar and pestle for several hours to achieve a uniform fine mixture, which is critical for promoting complete reaction and ensuring phase homogeneity.

The homogenized mixtures were then pressed into cylindrical pellets approximately 12 mm in diameter and 2 mm thick using a uniaxial hydraulic press. These pellets were subjected to multiple calcination steps in the temperature range of 700 °C to 1200 °C, each lasting around 20 hours, with intermediate grindings to enhance reaction completeness and improve crystallinity. These thermal treatments facilitate diffusion of the cations and oxygen, allowing for the formation of the desired layered perovskite structure. The general chemical reaction involved in the formation of LASMO *via* the solid-state route can be expressed as:(1 − *x*)La_2_O_3_ + _*x*_Ag_2_CO_3_ + SrCO_3_ + 2MnO_2_ → 2La_1−*x*_Ag_*x*_SrMn_2_O_5+*δ*_ + 2CO_2_↑

This equation represents the solid-state formation of the target compound, where La^3+^, Ag^+^, and Sr^2+^ occupy the A-site positions, while Mn ions are at the B-site of the layered perovskite structure. The oxygen non-stoichiometry (*δ*) accounts for any excess oxygen due to partial oxidation or reduction during heating, especially considering the variable valence states of Mn. After the final sintering step, the synthesized samples were characterized by X-ray diffraction (XRD) using powdered specimens to confirm phase purity, determine the crystal structure, and extract lattice parameters. These measurements ensure that the final products are structurally homogeneous and crystallize in the expected phase before proceeding to surface and magnetic analyses.

### 2.2. Sample characterization

To comprehensively evaluate the structural, microstructural, and magnetic properties of the synthesized LASMO compounds (*x* = 0.1 and 0.2), a combination of advanced characterization techniques was employed. X-ray diffraction was used as the primary method for structural analysis. Room-temperature measurements were performed using an X'Pert MPD Philips diffractometer equipped with a CuKα radiation source (*λ* = 1.54060 Å). The XRD data were collected over a wide 2*θ* range with a fine step size and long counting time to ensure high-resolution data suitable for phase identification, crystal structure confirmation, and lattice parameter refinement. These analyses allow the detection of any secondary phases, identification of the space group, and assessment of how Ag substitution at the A-site influences the orthorhombic perovskite structure. Rietveld refinement can be further applied to extract detailed structural parameters and quantify phase purity.

The surface morphology and grain size distribution were investigated using Scanning Electron Microscopy with a TESCAN Vega 3 system. The powders were dispersed and mounted on conductive stubs and then coated with a thin layer of carbon using a vacuum sputter coater to eliminate surface charging under the electron beam and to enhance image clarity. SEM imaging provided insights into the microstructural features of the samples, including grain connectivity, porosity, and texture, all of which influence the material's magnetic and transport properties. Any changes in grain morphology due to varying Ag content can also be correlated with magnetic performance. To explore the magnetic behavior, temperature-dependent magnetization measurements were conducted using a Superconducting Quantum Interference Device (SQUID) magnetometer, one of the most sensitive tools for detecting magnetic signals. The magnetization *M*(*T*) was recorded over the temperature range 5 K to 400 K under an applied magnetic field of 0.05 T. This range covers both low-temperature ordering phenomena and the high-temperature ferromagnetic–paramagnetic (FM–PM) transition, allowing accurate determination of the Curie temperature (*T*_p_) and other magnetic transition points. The derivative of the *M*(*T*) curve was analyzed to pinpoint *T*_p_ more precisely. Additionally, from these magnetization data, the magnetocaloric effect (MCE) was evaluated through the calculation of the magnetic entropy change (Δ*S*_m_) using the Maxwell relation. The corresponding relative cooling power (RCP) values were derived to assess the efficiency and applicability of these materials in magnetic refrigeration technologies.

## 3. Results and discussion

### 3.1. Qualitative analysis

**Fig. 1:**
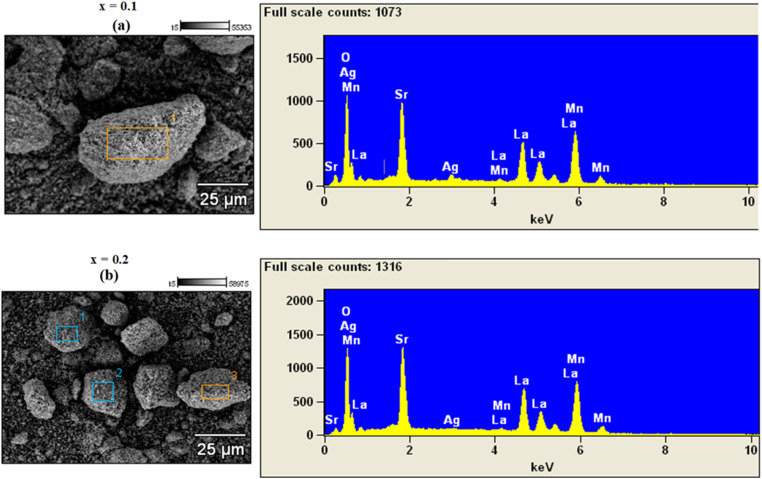
Spectra obtained by EDS analysis and Grain morphology in a powder of La_1−*x*_Ag_*x*_SrMn_2_O_5+*δ*_ ((a) *x* = 0.1 and (b) *x* = 0.2).

Qualitative elemental analysis of the synthesized samples (*x* = 0.1 and 0.2) was carried out using Energy Dispersive X-ray Spectroscopy (EDX), a technique that detects characteristic X-rays emitted from a sample surface when excited by an electron beam, allowing for the identification of constituent elements. As shown in Fig. 1, both samples exhibit EDX spectra consistent with the expected elemental composition based on the starting precursors. For *x* = 0.1 (Fig. 1a), the spectrum obtained from region 1 shows clear peaks for La (lanthanum), Ag (silver), Sr (strontium), Mn (manganese), and O (oxygen), indicating successful incorporation of all target elements. Similarly, the sample, *x* = 0.2 (Fig. 1b), with EDX data from region 3, reveals the same elemental peaks, further confirming consistent composition across different synthesis batches. The relatively similar spectral profiles and peak intensities between both samples suggest uniform elemental distribution and successful synthesis without contamination. These results collectively confirm that the intended multi-element composition was effectively achieved in both samples, validating the reliability and reproducibility of the synthesis process.

### 3.2. Morphological study

**Fig. 2:**
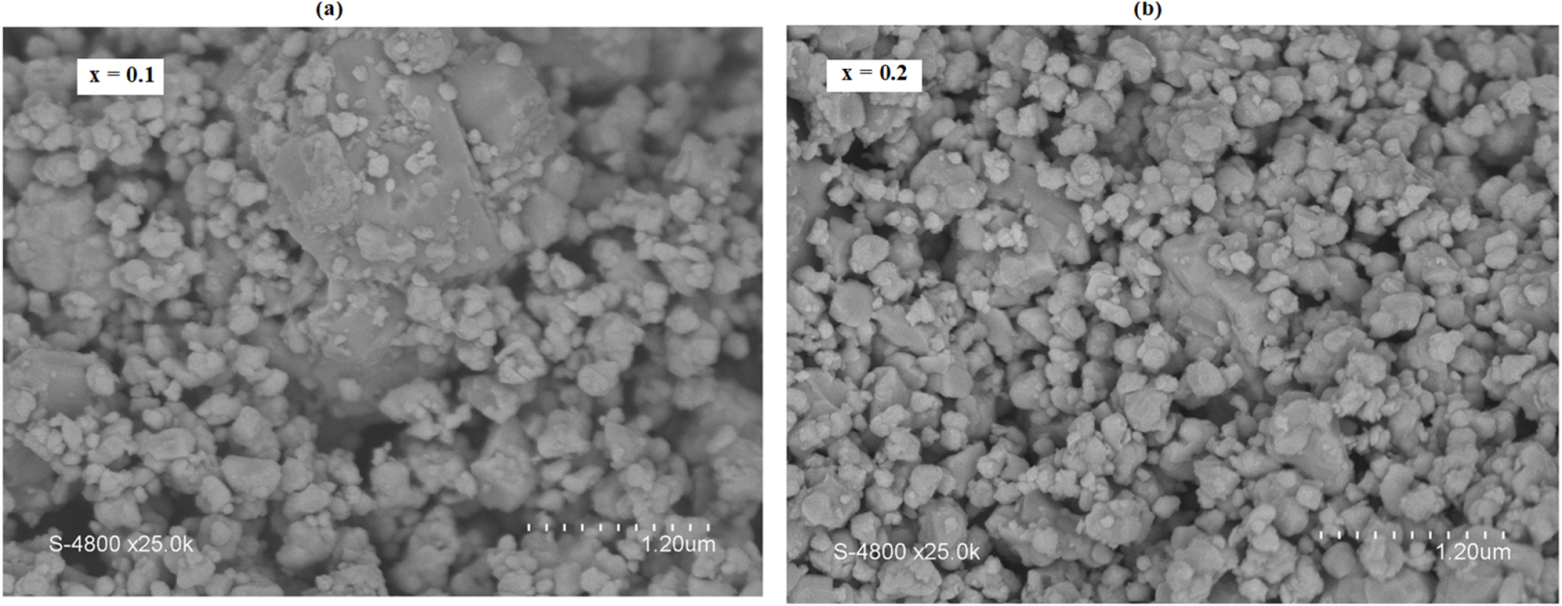
SEM micrographs of the LASMO series: (a and b) respectively for compositions *x* = 0.1 and *x* = 0.2.

The morphological characteristics of the LASMO samples were investigated using SEM, a high-resolution technique that reveals surface features and particle distribution. Fig. 2 shows SEM micrographs of the silver-doped samples with compositions *x* = 0.1 (Fig. 2a) and *x* = 0.2 (Fig. 2b), both imaged at the same magnification for direct comparison. The micrographs reveal that the samples are composed of densely packed agglomerates of crystallites, suggesting a compact and interconnected microstructure, which is favorable for enhanced transport properties.^137^ Both compositions exhibit relatively uniform morphology, with consistently distributed grains across the observed areas. Notably, the silver-doped samples display slightly larger grain sizes than the undoped LaSrMn_2_O_5_ parent compound, which typically shows finer particles. Based on SEM image analysis, the average grain sizes were estimated to be around 258 nm for the *x* = 0.1 sample and 238 nm for the *x* = 0.2 sample, respectively. These subtle variations in grain size may be attributed to the influence of Ag doping on the growth kinetics during the synthesis process, potentially promoting grain coarsening at lower doping levels.

### 3.3. Structural study by X-ray diffraction

**Fig. 3:**
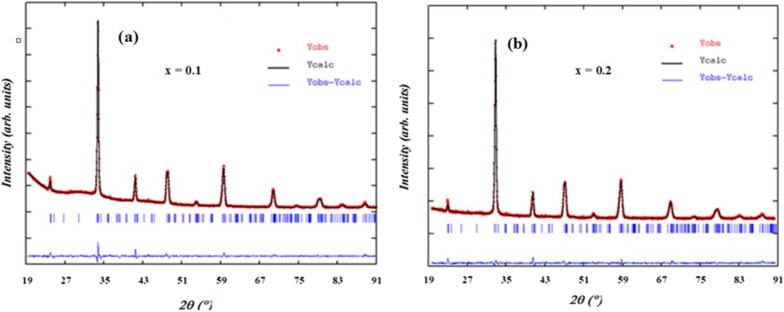
Calculated, observed, and difference X-ray diffraction patterns for La_1−*x*_Ag_*x*_SrMn_2_O_5+*δ*_ ((a) *x* = 0.1 and (b) *x* = 0.2).

X-ray diffraction patterns of LASMO compounds with doping levels *x* = 0.1 and 0.2 were collected at ambient temperature across a 2*θ* range of 20° to 91°, using a step size of 0.016°. The measurements were performed with Cu Kα radiation (*λ* = 1.54051 Å) as the X-ray source. Structural refinement of the recorded diffraction data was carried out using the Rietveld method implemented in the FullProf software suite.^138^ The XRD analysis confirmed that all samples crystallize in a single-phase orthorhombic structure, consistent with the *Pnma* space group. During refinement, atomic positions and isotropic displacement parameters were incrementally optimized. However, the thermal vibration parameters for oxygen atoms could not be accurately determined due to limitations inherent in X-ray scattering from light elements. The refinement process yielded a strong correlation between the experimental and calculated diffraction patterns, as illustrated in Fig. 3. The refined structural parameters, including lattice constants, atomic coordinates, and residual factors (*R*_B_, *R*_F_, and *χ*^2^), are summarized in Table 1, indicating good fit quality and structural consistency across the series.

**Table 1:** Structural parameters of La_1−*x*_Ag_*x*_SrMn_2_O_5+*δ*_ (*x* = 0.1 and *x* = 0.2) compounds obtained as a result of structural refinement by the Rietveld method

Samples	*a* (Å)	*b* (Å)	*c* (Å)	*V* (Å^3^)	*R*_B_ (%)	*R*_F_ (%)	*χ* ^2^
*x* = 0.1	5.450(5)	15.410(2)	5.495(7)	462.110(1)	4.6	1.4	1.5
*x* = 0.2	5.456(1)	15.409(5)	5.497(5)	462.206(5)	3.8	5.2	1.8

The incorporation of silver ions in place of lanthanum does not induce a structural phase transition in the LASMO compounds; however, it does significantly influence the lattice parameters. The observed changes in lattice constants with increasing Ag content can primarily be attributed to differences in ionic radii. As silver concentration increases, the lattice parameters *a* and *c* exhibit an upward trend, while parameter *b* decreases slightly. This anisotropic behavior results in an overall increase in the unit cell volume as the Ag substitution level rises. While the enlargement of the lattice can be partially explained by the larger ionic radius of Ag^+^ (1.42 Å) compared to La^3+^ (1.36 Å),^139^ additional factors must be considered. Notably, silver doping may also influence the Mn valence state by increasing the concentration of Mn^4+^ ions, because of Ag^+^ substituting La^3+^ at the A-site. This compensatory increase in Mn^4+^ alters the Mn^3+^/Mn^4+^ ratio, which helps maintain charge neutrality. Since Mn^3+^ ions contribute more strongly to Jahn–Teller distortions than Mn^4+^, changes in their relative abundance can subtly influence lattice dimensions and local structural distortions. It is worth noting that the lattice parameter *b* values reported here (∼15.41 Å) are approximately double the conventional *Pnma* cell dimension (typically ∼7.7 Å). This is due to the use of a doubled unit cell setting along the *b*-axis, which is commonly employed for layered perovskite structures and allows for the accommodation of potential superstructure effects, such as cation ordering, oxygen-vacancy modulation, or A-site layer alternation. This approach has been widely used in structurally similar manganites and is justified here by the observed reflection splitting and satisfactory Rietveld refinement indicators. It should be emphasized that this unit cell doubling along the *b*-axis does not indicate the formation of a double perovskite structure (A_2_BB′O_6_ type), as no B-site ordering or structural symmetry change was observed in the XRD patterns. X-ray powder diffraction is also an effective technique for determining the average crystallite size, often referred to as the coherent domain size, as well as for assessing lattice defects through analysis of peak broadening. Additionally, the average grain size of the material was estimated using the XRD peaks and the Scherer formula.^140^1
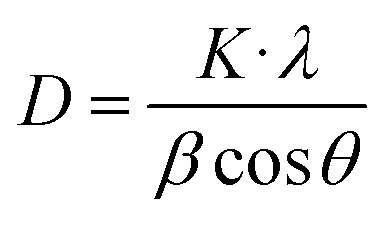
In this equation, *K* is a constant (0.89), *D* represents the average crystallite size, *λ* denotes the X-ray wavelength (1.54051 Å), *β* is the full width at half maximum (FWHM) of the diffraction peak, and *θ* is the Bragg diffraction angle corresponding to that peak. The calculated average crystallite sizes (*D*) were found to be 31.9 nm for the sample with *x* = 0.1 and 28.2 nm for *x* = 0.2, indicating that an increase in Ag content leads to a reduction in crystallite size. It is noteworthy that the particle sizes determined by SEM are larger than these crystallite dimensions. This discrepancy suggests that each particle observed under SEM comprises multiple crystallites, likely aggregated due to internal interactions or defects such as vacancies and dislocations.

### 3.4. Magnetic properties

Magnetic measurements as a function of temperature were carried out using a SQUID magnetometer. An external magnetic field of 0.05 T was applied while recording data over a temperature range from 5 K to 400 K. From these measurements, the ferromagnetic-to-paramagnetic transition temperature (*T*_C_) was determined by analyzing the derivative curves (d*M*/d*T*) as a function of temperature under the applied field. The observed width of this magnetic transition is influenced by the magnitude of the applied field; theoretically, *T*_C_ is defined as the temperature at which spontaneous magnetization emerges in the absence of an external field. However, because a field must be applied to obtain a measurable signal, it inevitably induces a magnetic moment above the intrinsic transition temperature, resulting in a broader transition profile. To balance these effects, we selected a field strength of 0.05 T sufficiently low to minimize transition broadening while still providing a significant magnetization signal.

**Fig. 4:**
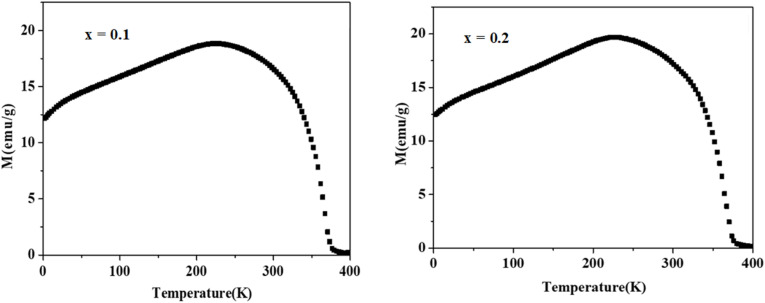
Variation of the magnetization as a function of temperature for *x* = 0, *x* = 0.1 and *x* = 0.2.

**Fig. 5:**
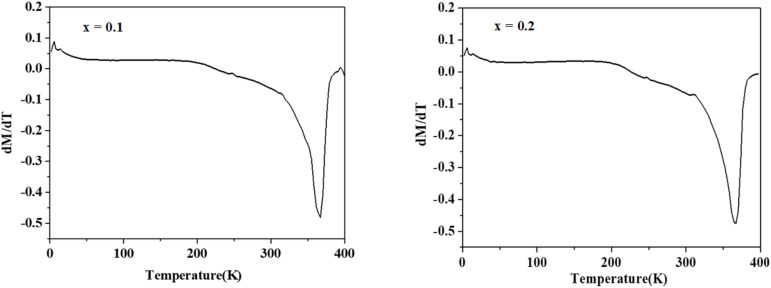
Variation of the derivative of the magnetization (d*M*/d*T*) as a function of the temperature, of the compounds La_1−*x*_Ag_*x*_SrMn_2_O_5+*δ*_ for *x* = 0.1 and 0.2.

**Fig. 6:**
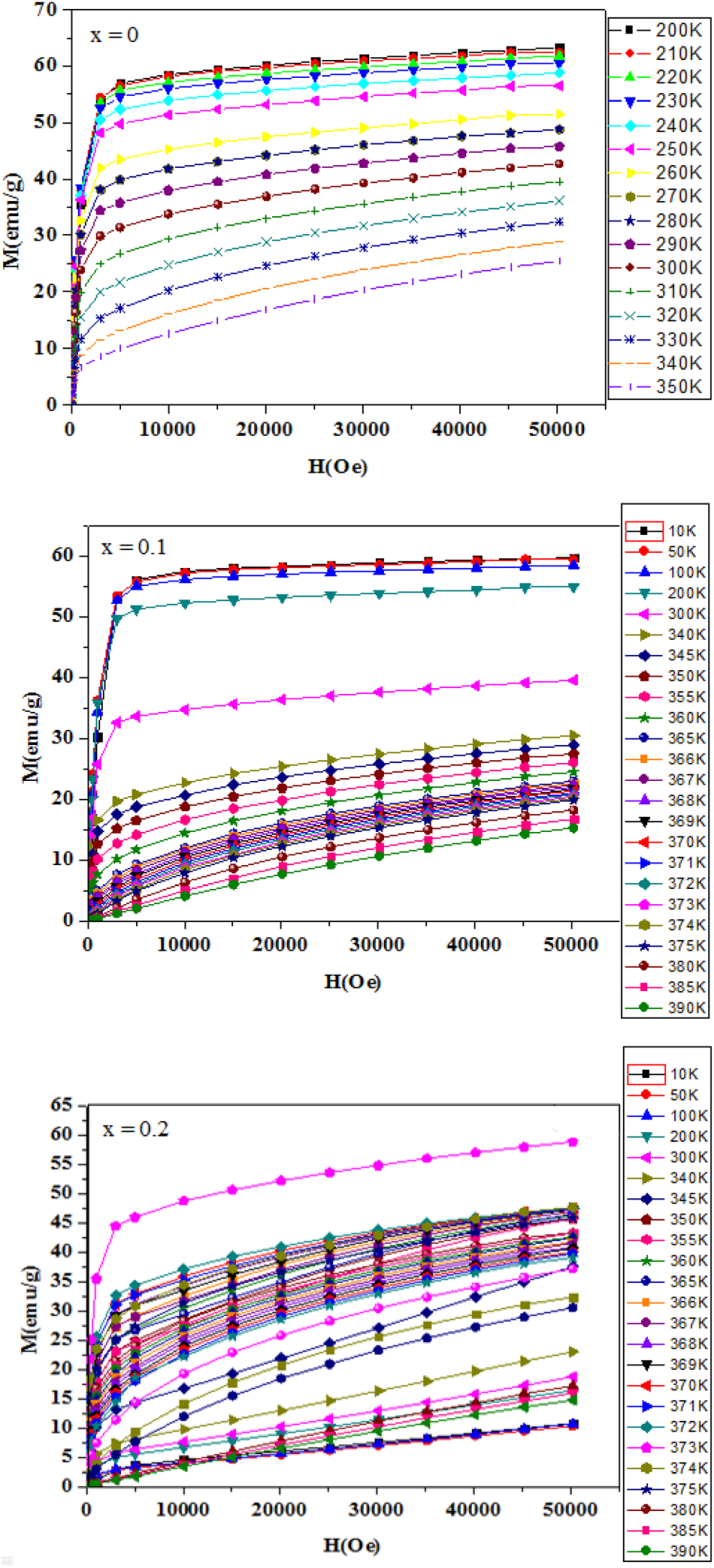
Magnetization *versus* magnetic applied field at various temperatures for the samples La_1−*x*_Ag_*x*_SrMn_2_O_5+*δ*_ with *x* = 0, *x* = 0.1 and *x* = 0.2.

To investigate the magnetic behavior of our samples, magnetization measurements were conducted as a function of temperature. Fig. 4 illustrates the thermal evolution of magnetization under an applied magnetic field of 0.05 T for La_1−*x*_Ag_*x*_SrMn_2_O_5+*δ*_ compounds with *x* = 0.1 and 0.2. As the temperature decreases, the magnetization curves clearly indicate a transition from the paramagnetic to the ferromagnetic state, with transition temperatures around 365 K, 366 K, and 367 K. The precise *T*_C_ marking the shift from the ferromagnetically ordered phase to the paramagnetic phase was determined from the derivative curves of magnetization with respect to temperature, as shown in Fig. 5. Notably, our time-dependent magnetization measurements reveal that *T*_C_ remains essentially constant across the different compounds. Interestingly, at low temperatures, a decline in magnetization is observed, which has been attributed to the formation of antiferromagnetic domains and/or the emergence of a charge-ordered phase. Additionally, Fig. 6 displays the *M*(*H*) isotherms for LASMO samples (*x* = 0, 0.1, and 0.2), recorded over a temperature range of 10 to 390 K with applied magnetic fields varying from 0 to 5 T.

The *M*(*H*) isotherms further verify the ferromagnetic nature of the samples at low temperatures. For temperatures below *T*_C_, the magnetization increases sharply and quickly reaches saturation under only a weak applied field. In contrast, when *T* exceeds *T*_C_, the material exhibits paramagnetic behavior, resulting in nearly linear magnetization curves as the field varies. The saturation magnetization values are approximately 5.4 *μ*_B_ per Mn for *x* = 0.1 and 4.2 *μ*_B_ per Mn for *x* = 0.2 (see Fig. 6). This reduction in saturation magnetization with higher Ag content is likely due to an enhanced presence of antiferromagnetic or charge-ordered states, which favor antiferromagnetic coupling at low temperatures.

**Fig. 7:**
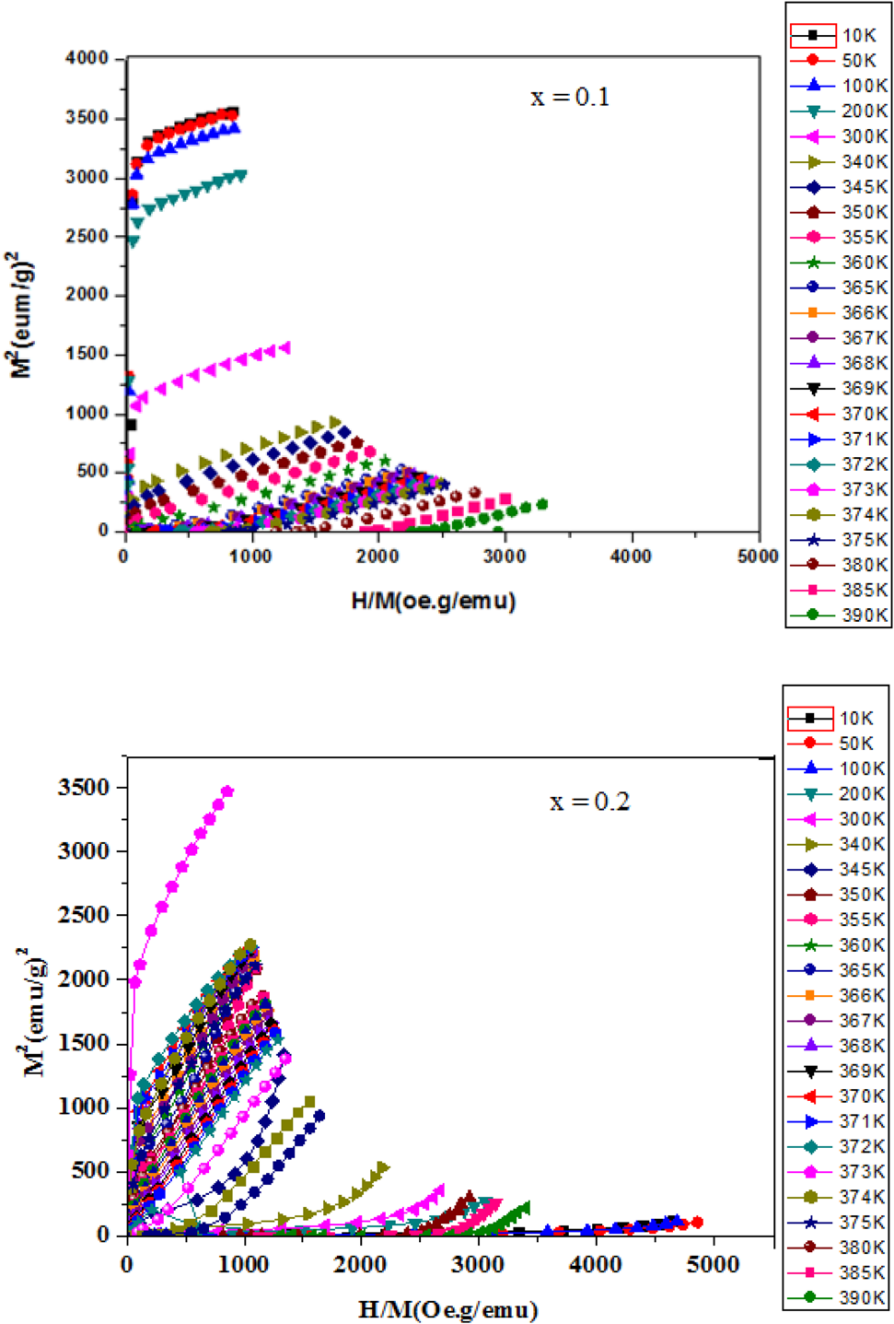
Arrott curves for La_1−*x*_Ag_*x*_SrMn_2_O_5+*δ*_ with *x* = 0, *x* = 0.1 and *x* = 0.2.

Fig. 7 shows a typical curve of the variation of *M*^2^*versus H*/*M* (Arrott curves) for the compound LASMO (*x* = 0.1 and *x* = 0.2). We notice that, for *T* < *T*_C_, all samples present isotherms with positive slopes which implies that the ferromagnetic–paramagnetic transition is of the second order. The Arrott curves also allow to determine the Curie temperature from the isotherm passing through the origin. The value of the Curie temperature *T*_C_ deduced from these Arott curves is very close to that deduced from the *M*(*T*) curve. The observed nanometric grain sizes are expected to affect the magnetic behavior of the LASMO samples. Reduced grain dimensions can lead to enhanced surface to volume ratios, which in turn increase the influence of surface spin disorder and grain boundary effects. These factors can suppress long-range ferromagnetic ordering, contributing to the observed decrease in saturation magnetization with increasing Ag content. Additionally, nanoscale effects may promote magnetic inhomogeneities or spin frustration, which could also explain the subtle low-temperature anomalies suggestive of competing magnetic interactions. Therefore, both chemical doping and nanoscale morphology likely act in concert to shape the overall magnetic response of these compounds. In addition, Ag^+^ substitution is expected to increase Mn^4+^ concentration and modify the Mn^3+^/Mn^4+^ ratio, the Curie temperature remains nearly unchanged across the doping range. This could be due to a compensation between increased hole doping and structural effects such as lattice distortions or changes in bond angles, which moderate the net effect on double exchange interactions. Further investigation, including direct oxygen stoichiometry measurements and electronic structure analysis, is necessary to fully understand this magnetic stability.

### 3.5. Study of the magnetocaloric effect

Magnetic entropy, which plays a crucial role in the magnetocaloric effect (MCE), can be determined from magnetization isotherms recorded under varying applied magnetic fields. According to classical thermodynamic principles, the magnetic entropy is linked to magnetization (*M*), external magnetic field (*μ*_0_*H*), and absolute temperature (*T*) through the Maxwell relation:^141,142^2
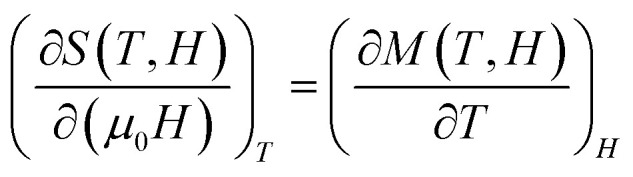


To avoid the challenges of direct adiabatic measurements, we derived magnetic entropy change (Δ*S*_m_) from isothermal magnetization data. The entropy change, corresponding to a field variation from 0 to *H*_max_, is computed using:3
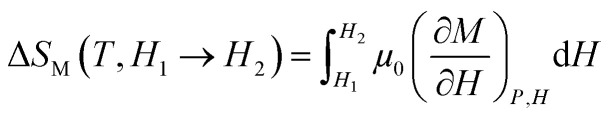


The MCE refers to the reversible thermal change observed when a magnetic material undergoes a transition from an ordered ferromagnetic (FM) to a disordered paramagnetic (PM) state. Near the Curie temperature (*T*_C_), this leads to a peak in magnetic entropy change.

**Fig. 8:**
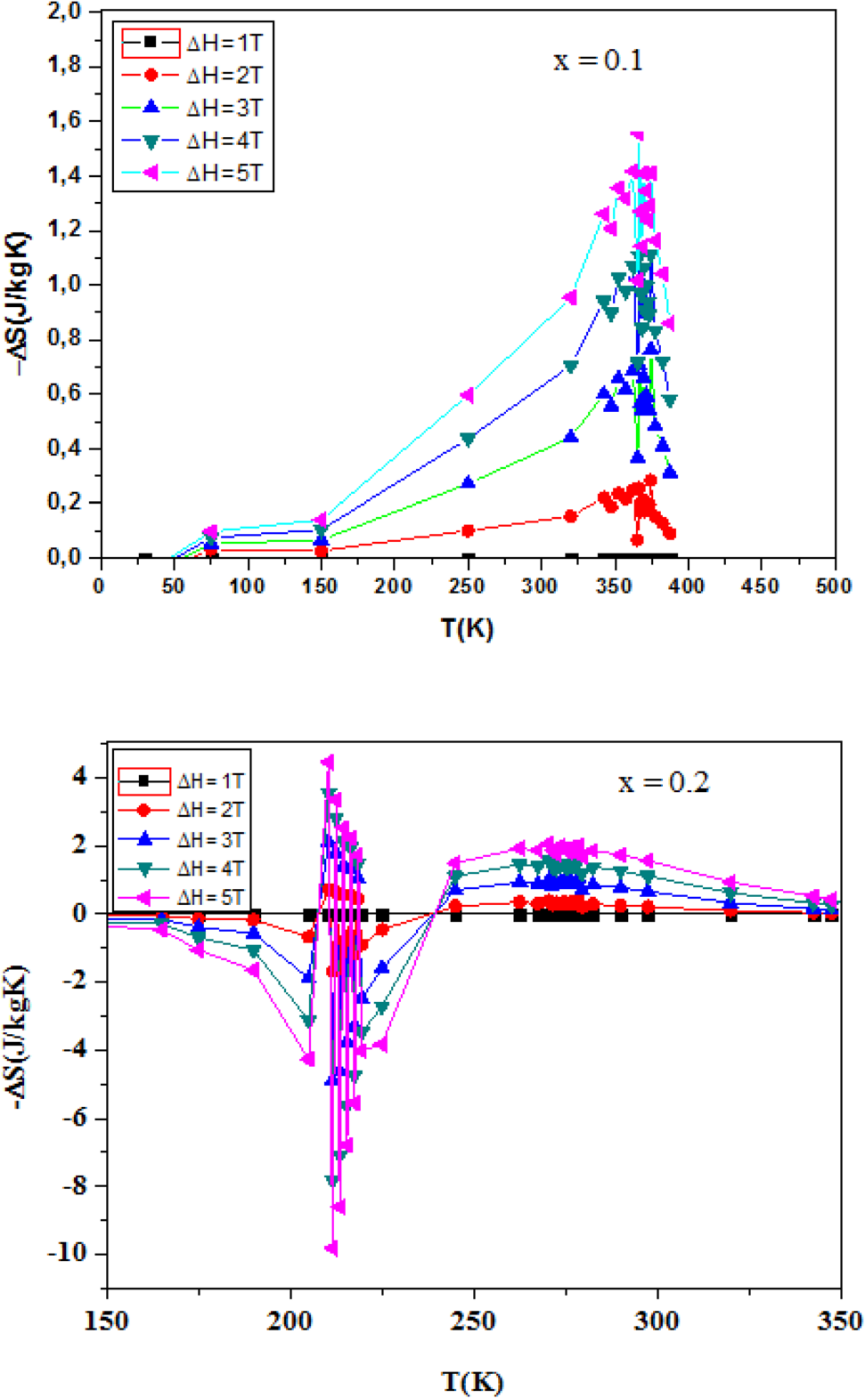
Variation of the magnetic entropy as a function of the temperature and the magnetic field of the compounds La_1−*x*_Ag_*x*_SrMn_2_O_5+*δ*_ (*x* = 0.1 and *x* = 0.2).

Fig. 8 illustrates the temperature dependence of the absolute magnetic entropy change |Δ*S*_m_| for La_1−*x*_Ag_*x*_SrMn_2_O_5+*δ*_ compounds with *x* = 0.0, 0.1, and 0.2, under different applied magnetic fields. For comparison, the undoped parent compound (*x* = 0) was also analyzed under the same conditions. The peak value of Δ*S*^max^_M_ is observed near *T*_C_ and increases with field strength. The results show that Δ*S*^max^_M_ increases systematically with Ag content: 1.28 J kg^−1^ K^−1^ for *x* = 0, 1.55 J kg^−1^ K^−1^ for *x* = 0.1, and 2.07 J kg^−1^ K^−1^ for *x* = 0.2 under an applied field of 5 T. These values are relatively high compared to other manganites, indicating enhanced fluctuations in magnetic order near the FM–PM transition.

While the value of Δ*S*^max^_M_ is an important parameter, it does not fully characterize a material's refrigeration efficiency. The relative cooling power (RCP) which accounts for both the magnitude and breadth of the entropy change is equally crucial. RCP is calculated as:

4 RCP = |Δ*S*^max^_M_|*δT*^FWHM^

where *δT*_FWHM_ represents the width of the Δ*S*_M_ peak at half maximum.

Table 2 summarizes the Δ*S*^max^_M_, *δT*^FWHM^, and RCP values for the samples at 5 T. Interestingly, although the Ag-doped samples show higher Δ*S*^max^_M_ values, the undoped sample (*x* = 0) exhibits the highest RCP (177.12 J kg^−1^) due to its broader Δ*S*_m_ peak. This highlights a trade-off between entropy magnitude and working temperature range, both of which are critical for real-world refrigeration applications. Overall, the LASMO compounds demonstrate promising MCE properties and tunability *via* silver substitution. These observations suggest that Ag substitution not only enhances the magnetic entropy change but also modifies the temperature span over which the effect occurs, likely due to alterations in the Mn^3+^/Mn^4+^ ratio and exchange interactions. The broader Δ*S*_m_ peak observed in the undoped sample may be indicative of more gradual magnetic transitions, while the sharper, higher peaks in Ag-doped compositions reflect stronger but narrower thermal responses. This tunability of MCE characteristics *via* compositional control offers valuable flexibility for optimizing materials based on specific operational requirements, such as compactness, thermal stability, or operating temperature range in practical systems. Moreover, the relatively high RCP and |Δ*S*_m_| values across all compositions underscore the robustness of the layered structure in sustaining significant magnetothermal responses.

**Table 2:** RCP values for La_1−*x*_Ag_*x*_SrMn_2_O_5+*δ*_ (*x* = 0, 0.1 and *x* = 0.2) samples

Composition	|Δ*S*^max^_M_| (J kg^−1^ K^−1^)	*δT* ^FWHM^ (K)	RCP (J kg^−1^)
LaSrMn_2_O_5+*δ*_	1.28	136.5	174.72
La_0.9_Ag_0.1_SrMn_2_O_5+*δ*_	1.55	106.1	164.455
La_0.8_Ag_0.2_SrMn_2_O_5+*δ*_	2.07	72.5	150.207

## 4. Conclusion

The La_1−*x*_Ag_*x*_SrMn_2_O_5+*δ*_ samples were successfully synthesized *via* the solid-state reaction method and systematically characterized through structural, morphological, and magnetic analyses. X-ray diffraction, combined with Rietveld refinement, confirmed that all compounds crystallize in a single-phase orthorhombic structure with space group *Pnma*, without any detectable structural phase transition upon Ag substitution. SEM analysis revealed densely packed crystallites with slight grain coarsening in Ag-doped samples. Magnetic measurements showed that all compositions undergo a second-order paramagnetic–ferromagnetic (PM–FM) transition.

The saturation magnetization was found to decrease with increasing Ag content, while the magnetic entropy change (−Δ*S*_m_) increased, accompanied by a reduction in relative cooling power (RCP). These trends are consistent with enhanced double exchange interactions and altered Mn valence distribution due to Ag incorporation. While the oxygen non-stoichiometry (*δ*) was not determined in this study, it is anticipated to influence the Mn^3+^/Mn^4+^ ratio and, consequently, the material's magnetic and electronic behavior. Future investigations will focus on quantifying *δ* to better understand its impact on the structure of property relationships in these systems. Overall, the results suggest that Ag-doped LASMO compounds possess promising magnetocaloric properties and could serve as potential candidates for magnetic refrigeration applications.

## Author contributions

Sonia Soltani: conceptualization, methodology, supervision, writing – original draft. Mokhtar Hjiri: experimental design, data curation, formal analysis, writing – review & editing. Abdullah M. Aldukhayel: investigation, resources, validation. Manel Essid: characterization (XRD, SEM), data interpretation. Anouar Jbeli: magnetic measurements, data analysis. Nouf Ahmed Althumairi: funding acquisition, project administration, writing review & editing. All authors have read and agreed to the published version of the manuscript.

## Conflicts of interest

The authors declare that they have no conflict of interest related to this work.

## Data Availability

The data that support the findings of this study are available from the corresponding author upon reasonable request.
